# Bacterial Lectin FimH and Its Aggregation Hot-Spots: An Alternative Strategy against Uropathogenic *Escherichia coli*

**DOI:** 10.3390/pharmaceutics15031018

**Published:** 2023-03-22

**Authors:** Georgia I. Nasi, Konstantina I. Georgakopoulou, Marilena K. Theodoropoulou, Nikos C. Papandreou, Evangelia D. Chrysina, Paraskevi L. Tsiolaki, Vassiliki A. Iconomidou

**Affiliations:** 1 Section of Cell Biology and Biophysics, Department of Biology, School of Sciences, National and Kapodistrian University of Athens, 15701 Athens, Greece; 2 Institute of Chemical Biology, National Hellenic Research Foundation, 11635 Athens, Greece

**Keywords:** FimH, lectin, type I fimbriae, aggregation-prone regions, prediction, UTIs, UPEC

## Abstract

Type I fimbriae are the main adhesive organelles of uropathogenic *Escherichia coli* (UPEC), consisting of four different subunits. Their component with the most important role in establishing bacterial infections is the FimH adhesin located at the fimbrial tip. This two-domain protein mediates adhesion to host epithelial cells through interaction with terminal mannoses on epithelial glycoproteins. Here, we propose that the amyloidogenic potential of FimH can be exploited for the development of therapeutic agents against Urinary Tract Infections (UTIs). Aggregation-prone regions (APRs) were identified via computational methods, and peptide-analogues corresponding to FimH lectin domain APRs were chemically synthesized and studied with the aid of both biophysical experimental techniques and molecular dynamic simulations. Our findings indicate that these peptide-analogues offer a promising set of antimicrobial candidate molecules since they can either interfere with the folding process of FimH or compete for the mannose-binding pocket.

## 1. Introduction

Urinary Tract Infections (UTIs) are one of the most common bacterial infectious diseases worldwide [1], and it is estimated that 40–50% of women and 5% of men will develop a UTI at least once in their lifetime [2]. Bacterial infections of the urinary tract (UT) may be caused by an array of organisms [3], with uropathogenic *Escherichia coli* (UPEC) being the primary cause of up to 70–95% of UTIs [4,5]. The main treatment for uncomplicated UTIs is a short course of antibiotics. However, the emergence of antibiotic-resistant UPEC strains and the latency in the development of new antibiotics highlight the dire need for novel and effective drugs against UPEC and other pathogenic *E. coli* strains [6,7]. An attractive target to treat UTIs is the mannose-specific fimbrial adhesin of *E. coli*, named FimH.

FimH adhesin is located at the tip of *E. coli* type I fimbriae [8,9,10], which are long filamentous, adhesive organelles [11] expressed on the surface of most *E. coli* strains, as well as in most members of the *Enterobacteriaceae* family [10]. They play a crucial role in the establishment and formation of colonies in the bladder as they mediate the bacterial binding to glycoproteins bearing terminally exposed mannose [12]. More specifically, since the 1980s, it has been clear that uropathogenic strains of *E. coli* that carry these fimbriae are responsible for causing UTIs in humans [13,14]. Each fibril is composed of up to 3000 copies of the major structural subunit FimA, building a rigid and helically wound rod, as well as two minor subunits, namely FimF and FimG, and the adhesin FimH, forming the distal tip of the fibrillum [15].

FimH protein’s precursor is a 300-residue-long polypeptide chain, which, after having its signal peptide cleaved off, is composed of 279 residues [16]. FimH consists of two immunoglobulin-like domains connected by a flexible linker. The N-terminal lectin domain mediates the binding to the mannose ligand, while the C-terminal pilin domain anchors the adhesin to the fimbrial tip and regulates the conformation and, consequently, the function of the lectin domain [17,18]. Two main conformational states of the lectin domain are observed: one with a wide mannose-binding site (low affinity) and one with a narrow site around the ligand (high affinity) [18]. The low-affinity state of FimH is the result of the interaction between the lectin and the pilin domains. On the other hand, the separation of the two domains leads to a shift from the low- to the high-affinity state. FimH plays a pivotal role in establishing UTIs since it mediates the binding and subsequently the endocytosis of bacteria to host cells, where they are protected by the immune system and antibacterial drugs [8]. Furthermore, it has been shown that *E. coli* FimH mutants have an impaired ability to form the fimbriae, suggesting an essential role in its biogenesis [19,20]. Additionally, vaccination of primates and mice with FimH resulted in reduced bladder colonization [21]. Therefore, it can be firmly deduced that this protein is an attractive therapeutic agent for drug development of UTIs treatment.

The worrying problem of multidrug-resistant bacteria, as well as the critical role of FimH in type I fimbriae formation and bladder colonization, led us to exploit the amyloidogenic profile of FimH to design peptides-analogs capable of affecting its folding or function. We used AMYLPRED2, a computational tool that utilizes a consensus of 11 individual methods for predicting amyloid-forming regions [22], in order to identify aggregation-prone regions (APRs) in the FimH mature sequence. By analyzing the protein’s amino acid sequence, AMYLPRED2 can identify regions that are more likely to be involved in amyloid formation. The threshold used to predict a protein segment as an APR is n/2, where n is the number of algorithms used in the analysis (see Section 2.1). Out of the predicted APRs, four were chemically synthesized, and their self-aggregation process was tested with the aid of experimental biophysical techniques. Molecular Dynamics (MD) simulations were utilized to evaluate the affinity of the peptide-analogues for the FimH lectin’s mannose-binding pocket. Additionally, for each selected peptide-analogue, we calculated the number of times they occurred in three proteomes. The results show that some of the selected peptide-analogues can self-assemble into amyloid-like fibrils. Based on our findings, we propose that the self-aggregation properties of at least one of the peptide-analogues could be utilized to hinder the FimH folding process, leading to an inability to form type I fibrils and consequently treat UTIs.

## 2. Materials and Methods

### 2.1. Aggregation Propensity Analysis of FimH

The prediction algorithm AMYLPRED2 (http://biophysics.biol.uoa.gr/AMYLPRED2/, June 2021) [22] was used for the identification of APRs in mature FimH (UniProt AC: P08191 [23]). To analyze the protein sequence, we input it in FASTA format into AMYLPRED2—a consensus tool that integrates several properties to predict sequence amyloidogenicity. It utilizes eleven different prediction algorithms. To identify APRs, AMYLPRED2 employs a threshold of n/2, where n is the number of algorithms used each time. The threshold is rounded down to the previous integer, and any protein segment with a score above the threshold is predicted to be an APR. The results of the prediction indicate that the mature sequence of FimH, along its entire length, has several regions with an increased tendency to self-aggregate (Figure S1).

### 2.2. Peptide Design and Synthesis

**Figure 1:**
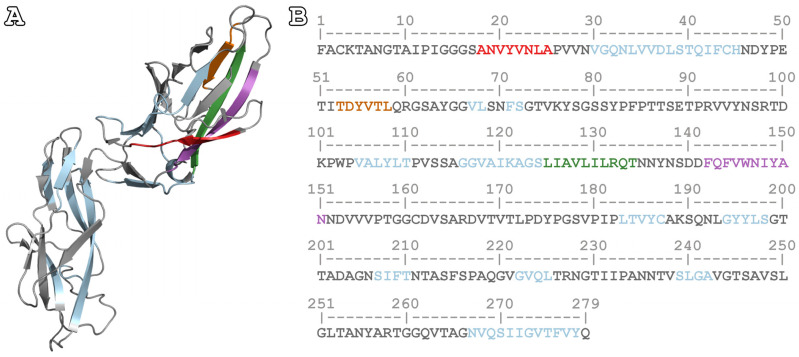
Native structure and mature sequence of FimH. (**A**) The native structure of FimH, in the absence of any ligand (PDB ID: 3JWN) [17]. The selected peptide-analogues for the experimental study are located in the lectin domain, close to the loops forming part of the mannose-binding site. (**Β**) The amino acid sequence of FimH. The predicted APRs have been highlighted with light blue, while the experimentally studied APRs have been marked in different colors. More specifically, the segments colored in red, orange, green and purple depict the APRs ^18^ANVYVNLA^25^, ^53^TDYVTL^58^, ^125^LIAVLILRQT^134^, and ^142^FQFVWNIYAN^151^, respectively. Segments with low aggregation propensity are shown in grey.

Considering that the FimH lectin domain has been the focus of interest for finding new therapies for UPECs since it mediates the attachment of bacteria to host cells, we opted to study only APRs found in this domain. More specifically, four APRs were selected, corresponding to one of the two β-sheets of this domain (Figure 1A). The exact sequences for the peptide-analogues were ^18^ANVYVNLA^25^, ^53^TDYVTL^58^, ^125^LIAVLILRQT^134^, and ^142^FQFVWNIYAN^151^ (Figure 1B).

All four peptide-analogues were synthesized and lyophilized by GeneCust Europe, France. The purity of all the synthesized peptide-analogues was higher than 98%, with the N- and C-terminal being free.

### 2.3. In Vitro Amyloid Fibril Formation

All four lyophilized peptide-analogues were dissolved in distilled water (pH 5.5) at a concentration of 10 mg/mL. The ^18^ANVYVNLA^25^ peptide-analogue was dissolved at concentrations ranging from 5 mg/mL to 10 mg/mL since it formed an incredibly dense gel at higher concentrations. Subsequently, the solutions of the peptide-analogues were incubated for 1 week at room temperature.

### 2.4. Negative Staining and Transmission Electron Microscopy (TEM)

Each peptide-analogue solution (5 μL) was applied to a glow-discharged 400-mesh and carbon-coated copper TEM grid for about 60 s, and the excess sample was blotted away with filter paper. The grid was then stained with a 5 μL drop of 2% (*w*/*v*) aqueous solution of uranyl acetate for another 60 s. The excess stain was removed by blotting with filter paper. The grid was allowed to air-dry for a few seconds and then examined using a Morgagni^TM^ 268 transmission electron microscope, operated at 80 kV. Digital acquisitions were performed with an 11 Mpixel side-mounted Morada CCD camera (Soft Imaging System, Muenster, Germany).

### 2.5. X-ray Diffraction from Oriented Protein Fibers

A 5 μL droplet of each peptide-analogue solution was placed between two aligned siliconized glass rods mounted on a glass slide, spaced approximately 2 mm apart and oriented horizontally, as collinearly as possible. Each sample was allowed to air-dry slowly at ambient temperature and humidity for approximately 30 min to form an oriented fiber suitable for X-ray diffraction. The X-ray diffraction patterns were collected using a SuperNova-Agilent Technologies X-ray generator equipped with a 135-mm ATLAS CCD detector and a 4-circle kappa goniometer (CuKα high-intensity X-ray micro-focus source, λ = 1.5418 Å), operated at 50 kV, 0.8 mA, installed at the Instruct-EL hub, Institute of Chemical Biology, National Hellenic Research Foundation, which is part of the national research infrastructure on structural biology Inspired. The oriented fiber sample was mounted onto the goniometer, and the specimen-to-film distance was set at 52 mm, with an exposure time of 400 s. Each X-ray diffraction pattern was initially viewed using the CrysAlisPro v. 171.40.67a software [24] and consequently displayed and measured with the aid of the iMosFLM v. 7.3.0 software [25].

### 2.6. Attenuated Total Reflectance Fourier-Transform Infrared Spectroscopy (ATR FT-IR) and Post-Run Spectra Calculations

Drops (3 μL) of the FimH peptide-analogue solutions were applied on a front-coated Au mirror plate (SpectRIM, Tienta Sciences, Inc., Indianapolis, IN, USA) and were left to air-dry slowly at ambient conditions until thin peptide-containing films were formed. IR spectra of these films were obtained at a resolution of 4 cm^−1^, utilizing an IR microscope (IRScope II, BrukerOPTICS, Bruker Optik GmbH, Ettlingen, Germany) equipped with a Ge ATR objective lens (20×) and attached to an FT spectrometer (Equinox 55, BrukerOPTICS, Bruker Optik GmbH, Ettlingen, Germany). In total, ten 32-scan spectra were collected from each sample and averaged to improve the Sound/Noise (S/N) ratio [26]. The spectra are shown in the absorption (A) mode after correction for the wavelength dependence of the penetration depth (pd ~ λ). Second derivatives were computed analytically using routines of the Bruker OPUS/OS2 software (Bruker Optik GmbH, Ettlingen, Germany), including smoothing over a 13 cm^−1^ range around each data point, performed by the Savitsky–Golay algorithm [27]. The data was visualized using OriginPro 7 (OriginLab Corporation, Northampton, MA, USA). Absorption band maxima were determined from the minima in the second derivative of the corresponding spectra.

### 2.7. Congo Red Birefringence Assay

A 3 μL drop of each peptide-analogue solution was applied to glass slides and left to air-dry at room temperature and humidity until a thin film on top of the slide plate was produced. Subsequently, the films were stained with a 1% (*w*/*v*) Congo Red solution in distilled water (pH 5.5) [28,29] for approximately 30 min. Excess stain was removed through several tap water washes, and the stained films were left to air-dry for approximately 10 min. The samples were observed under bright field illumination and between crossed polars, using a LeicaMZ7.5 polarizing stereomicroscope (Leica Camera AG, Weltzar, Germany), equipped with α Sony α6000 camera (Sony, Tokyo, Japan).

### 2.8. Molecular Dynamics (MD) Simulations

#### 2.8.1. Data Retrieval

Simulations were performed using a PDB structure of the FimH lectin domain (PDB ID: 1UWF) [30]. A 3D structure of butyl alpha-D-mannopyranoside—a D-mannose analogue, hereafter called DEG—retrieved from PubChem [31] (PubChem CID: 656941), while the structure of each peptide-analogue was generated through the “Builder” tool in PyMOL v. 2.5.0 [32]. In order to minimize unnecessary calculations during the simulation, we selected a FimH structure that contains only the lectin domain, which is responsible for the binding of the bacteria to the bladder epithelium glycoproteins [30]. At the same time, the lectin domain at this PDB entry is in the high-affinity conformational state for the D-mannose, allowing us to observe if the peptide-analogue can compete for the mannose-binding pocket.

#### 2.8.2. Molecular Docking

In order to create the homo-oligomerization system of each peptide-analogue, the automated protein docking server ClusPro [33,34,35] was utilized. Specifically, the routine “Peptide docking” and its subroutine “multimer” were used to create our systems. The simulation boxes contained five (5) copies of each peptide-analogue in random conformations, previously shown to be an efficient number of copies for self-assembly simulations [36,37]. The generated clusters were evaluated with the balanced scoring scheme.

Structural models of the FimH lectin domain-DEG and FimH lectin domain-DEG-peptide-analogue complexes were predicted by the HADDOCK v.2.4 web server [38,39]. The HADDOCK score was used to rank and evaluate the generated clusters.

#### 2.8.3. MD Simulations

The four homo-oligomerization systems, the FimH lectin domain-DEG complex—used as a control—and the four FimH lectin domain-DEG-peptide-analogue complexes were subjected to MD simulations via GROMACS v. 2018.1 [40]. In the case of the homo-oligomerization simulations, the Amber ff99sb-ILDN force field [41] was used since it is considered more accurate for simulating unfolded peptides as well as the assembly of amyloid peptides [42,43]. On the other hand, for the FimH lectin domain-DEG-peptide-analogue complexes, the CHARMM36 protein force field was utilized since it is considered the most suitable for protein-ligand MD simulations since it includes parameters for a multitude of both biological and chemical molecules [44,45]. The systems were placed into a cubic unit cell, with a distance of 20 Å between each cell. The solvent was modeled using the TIP3P water model [46], and the systems were ionized using NaCl molecules to mimic neutral pH conditions. The systems underwent energy minimization using the steepest descent algorithm for a maximum of 2000 steps, followed by two stages of equilibration simulations with position restraints applied on the protein coordinates. The first equilibration was a 100 ps simulation in the canonical (NVT) ensemble to adjust the temperature at 310 K using the Berendsen thermostat [47]. The second equilibration was a 100 ps simulation in the isothermal-isobaric (NPT) ensemble to control pressure isotopically at 1.013 bar (1 atm), using the Berendsen weak coupling algorithm [48] and the Berendsen-thermostat at 310 K. Finally, at 310 K, the MD simulation was run for 500 ns for the homo-oligomerization systems and 350 ns for the lectin domain complexes, with position restraints removed. The LINCS algorithm [49] was used to model bond constraints, allowing for a time-step of 2 fs. Short-range non-bonded interactions were modeled using a twin-range cutoff at 0.8 nm, while long-range electrostatic interactions were modeled using the Particle Mesh Ewald (PME) method, with a Fourier grid spacing of 0.12 nm [50].

#### 2.8.4. Analysis of Simulation Results

The simulation results were analyzed using a variety of tools, including GROMACS utilities, DSSP [51], and PyMOL [32]. The pictures were generated with PyMOL. The acquired graphs were created using the R v. 4.1.2 statistical language and the ggplot2 v. 3.4.1 package in the integrated development environment RStudio.

### 2.9. Redundancy of the Selected Peptide-analogues

To analyze the redundancy of the four “aggregation-prone” sequences, the number of times they occurred in the *E. coli*, *Mus musculus*, and *Homo sapiens* proteome was calculated. Since single-point mutations still may promote self-assembly [52,53], zero-, one-, and two-amino acid mutations were taken into consideration. For the analysis, Python programming language was used to write a pattern-matching script using the proteomes of *E. coli* (UP000000625), *M. musculus* (UP000000589), and *H. sapiens* (UP000005640) from UniProt [23] in FASTA format.

## 3. Results

### 3.1. Aggregation Assays of FimH Peptide-Analogues

Our experimental findings reveal that three out of the four FimH peptide-analogues studied here can be characterized as amyloidogenic in vitro. The characterization of their aggregates as amyloid-like fibrils were based on the tinctorial characteristics for the identification of amyloid fibrils [54]. Specifically, TEM, X-ray diffraction from protein fibers, ATR FT-IR spectroscopy, and Congo Red staining were used to examine the amyloidogenic properties of FimH peptide-analogues.

**Figure 2:**
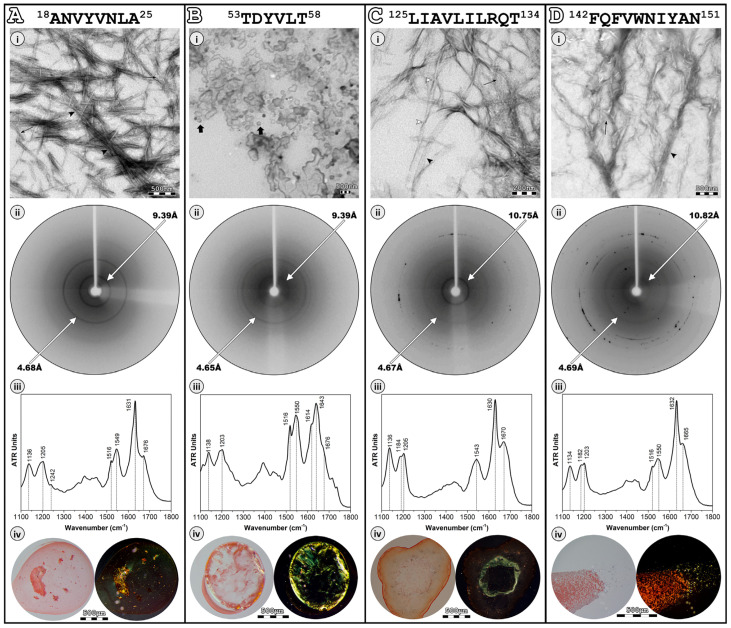
Experimental results of the four peptide-analogues (**A**–**D**). ^18^ANVYVNLA^25^ (**Ai**), ^125^LIAVLILRQT^134^ (**Ci**) and ^142^FQFVWNIYAN^151^ (**Di**) self-assemble into straight and unbranched amyloid-like fibrils, with a diameter of approximately 100 Å (black arrows), which tend to interact laterally forming straight and twisted ribbons (black and white arrowheads, respectively). ^53^TDYVTL^58^ (**Bi**) forms amorphous and spherical (flat black arrows) aggregates. All X-ray diffraction patterns (**Aii**–**Dii**) are indicative of the “cross-β” structure, displaying a reflection at approximately 4.7 Å and a reflection at 9.39 Å, 9.39 Å, 10.75 Å, and 10.82 Å, corresponding to the distance between consecutive β-strands and the distance between packed β-sheets, respectively. ATR FT-IR spectra (1100–1800 cm**^−^**^1^) produced from thin hydrated films of the four peptide-analogues (**Aiii**–**Diii**) confirm their β-sheet secondary structure. All peptide-analogues exhibit the characteristic apple–green birefringence (**Aiv**–**Div**) that amyloids typically exhibit.

To inspect the morphology of the self-assemblies formed from the four peptide-analogues, a sample from each peptide solution—after an incubation period of 1 week—was observed utilizing TEM after negative staining (uranyl acetate 2% (*w*/*v*)). Transmission electron micrographs show that peptide-analogues ^18^ANVYVNLA^25^, ^125^LIAVLILRQT^134^, and ^142^FQFVWNIYAN^151^ self-assemble into amyloid-like fibrils. These fibrous aggregates are straight and unbranched, with a diameter of approximately 100 Å (Figure 2, black arrows) and tend to interact laterally, forming straight bundles or flat ribbons of different diameters (Figure 2, black arrowheads). In the case of ^125^LIAVLILRQT^134^, twisted ribbons are also observed (Figure 2(Ci), white arrowheads), suggesting that the amyloid-like fibrils are flexible. On the other hand, ^53^TDYVTL^58^ forms amorphous aggregates, some of which have a more defined spherical shape and a diameter ranging between 150–350 nm (Figure 2(Bi), flat black arrows).

Oriented fibers by each peptide solution produced X-ray diffraction patterns that revealed that all peptide-analogues adopt well-ordered β-sheets. The X-ray diffraction patterns of ^18^ANVYVNLA^25^, ^125^LIAVLILRQT^134^, and ^142^FQFVWNIYAN^151^ (Figure 2(Aii–Dii))—the three amyloid-like fibril forming peptide-analogues—resemble the “cross-β” pattern, usually observed in other amyloidogenic proteins. Specifically, a reflection at approximately 4.7 Å is observed at all three patterns, corresponding to the interchain distance between hydrogen-bonded-strands. Moreover, a second strong equatorial reflection, appearing at 9.39 Å, 10.75 Å, and 10.82 Å for ^18^ANVYVNLA^25^, ^125^LIAVLILRQT^134^, and ^142^FQFVWNIYAN^151^ peptide-analogues, respectively, is ascribed to the variable packing distance between packed β-sheets. Even though ^53^TDYVTL^58^ exhibits decreased amyloidogenicity since it forms amorphous aggregates, its X-ray diffraction pattern reveals an unexpected β-sheet structure (Figure 2(Bii)). This should come as no surprise since other studies have shown that amorphous aggregates can also adopt the β-sheet structure [55,56]. The ring-like appearance of all the reflections is due to poor alignment of the oriented fiber constituent fibrils [57].

As additional structural evidence, thin hydrated fibril-containing films were studied with the aid of ATR FT-IR spectroscopy. The ATR FT-IR results confirmed the X-ray diffraction data, supporting the dominance of the β-sheet secondary structure. The IR spectra analysis revealed characteristic peaks at amide I and II that can be assigned to β-sheets (Table 1 and Figure 2(Aiii–Diii)).

It has been shown that amyloid fibrils bind the Congo Red dye with high affinity and display an apple–green birefringence when observed under crossed polars of a polarizing microscope [54]. Paradoxically, our results indicate that all four peptide-analogues bind the amyloid-specific Congo Red dye, as seen under bright field illumination in a polarizing microscope (Figure 2(Aiv–Div), left). When the polars are crossed, ^18^ANVYVNLA^25^ and ^125^LIAVLILRQT^134^ peptide-analogues exhibit the characteristic apple–green birefringence (Figure 2(Aiv,Civ), right). On the other hand, the ^142^FQFVWNIYAN^151^ peptide-analogue, despite forming a dense network of amyloid-like fibrils, presents apple–green birefringence only at the edges of the dyed fibril-containing film, while the center is mainly orange (Figure 2(Div), right). The tight packing of the fibrils may not allow the excess stain to be removed, leading to the orange birefringence observation. It is noteworthy to mention that orange birefringence has been considered in the past a positive indication for the identification of amyloid fibrils [58], implying that our results do not annul the potential amyloidogenic character of ^142^FQFVWNIYAN^151^. Finally, the ^53^TDYVTL^58^ peptide-analogue unexpectedly shows apple–green birefringence (Figure 2(Biv), right). It is possible that the Congo Red molecules were able to bind with a specific orientation that allows the observation of the apple–green birefringence, considering the well-ordered β-structure of this peptide-analogue [59].

### 3.2. Self-Oligomerization Simulations

**Figure 3:**
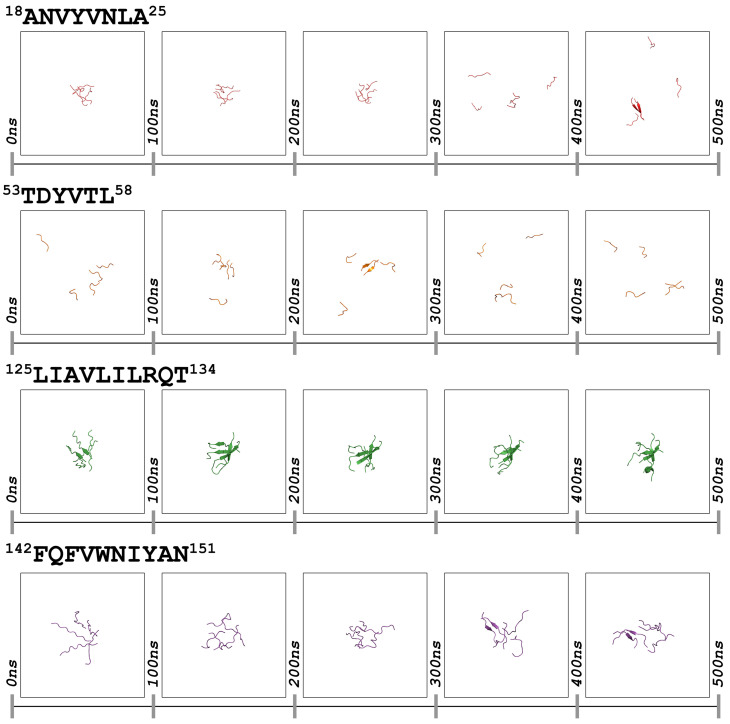
MD simulations of ^18^ANVYVNLA^25^, ^53^TDYVTL^58^, ^125^LIAVLILRQT^134^, and ^142^FQFVWNIYAN^151^ peptide-analogues. In every case, a solvent box containing five copies of each peptide-analogue was simulated for 500 ns. Peptide-analogues are shown as cartoons, utilizing PyMOL v.2.5.0 [32].

The self-assembly of the four peptide-analogues was evaluated computationally by performing MD simulations. In random configurations, five copies of each peptide-analogue were placed in a box with an aqueous solvent and simulated for 500 ns. A general view of these simulations shows that in all cases, the peptide-analogues diffuse freely in the simulation box, leading to numerous association and dissociation events (Figure 3). This finding is consistent with results from MD simulations of the early oligomerization events of the amyloidogenic peptide NH2-GNNQQNY-CH3, which showed that many association and dissociation events could take place on the nanosecond and microsecond scales [60].

Of the four homo-oligomerization systems, ^125^LIAVLILRQT^134^ peptide-analogue exhibited the most significant correlation properties, as it forms assemblies with β-structure characteristics from the beginning of the simulation, which are maintained throughout the whole simulation (Figure 3). Similar properties were observed for the ^18^ANVYVNLA^25^ and ^142^FQFVWNIYAN^151^ systems since both peptide-analogues form aggregates, but at a later stage compared to the ^125^LIAVLILRQT^134^ peptide-analogue (Figure 3). Comparing the two systems, the lifetime of the formed oligomers is slightly longer for the ^142^FQFVWNIYAN^151^ peptide-analogue in contrast to that of the ^18^ANVYVNLA^25^ peptide-analogue. On the other hand, all the aggregates observed in the simulations of the ^53^TDYVTL^58^ peptide-analogue were short-lived, dissociating within 5–10 ns of their formation (Figure 3). Although details about the aggregation mechanism of each peptide-analogue were not retrieved, the results show that, overall, the ^53^TDYVTL^58^ peptide-analogue exhibits limited aggregation potential compared to the other three peptide-analogues. This assumption is aligned with the experimental results. In terms of secondary structure, the complexes produced by the systems of ^18^ANVYVNLA^25^, ^125^LIAVLILRQT^134^, and ^142^FQFVWNIYAN^151^ adopted β-structure features and resembled aggregates previously observed in simulations of the amyloidogenic NH_2_-GNNQQNY-CH_3_ peptide [60].

### 3.3. Affinity for the Mannose-Binding Pocket

FimH binding to uroepithelial receptors can be inhibited by D-mannose and a variety of natural and synthetic saccharides that contain mannose-terminal residues [30,61,62]. Blocking the interaction of FimH with the receptor can prevent bacterial adhesion to the bladder uroepithelium and, therefore, infection [21,63]. In an effort to investigate the affinity of the four peptide-analogues against the mannose-binding pocket, considering their ability to self-aggregate and their location on the FimH lectin domain, MD simulations were performed. In these in silico experiments, each peptide-analogue and a D-mannose analogue (DEG) were docked between the mannose-binding loops (amino residues 46–54 and 136–140) [64]. It is noteworthy to mention that before carrying out the experiments for the complexes of the peptide-analogues and FimH lectin domain—DEG, a simulation of the lectin domain with the D-mannose analogue was performed. The thermodynamic simulation analysis demonstrated DEG as a stable interactor with the binding position throughout the 350 ns simulation (Figure S2).

**Figure 4:**
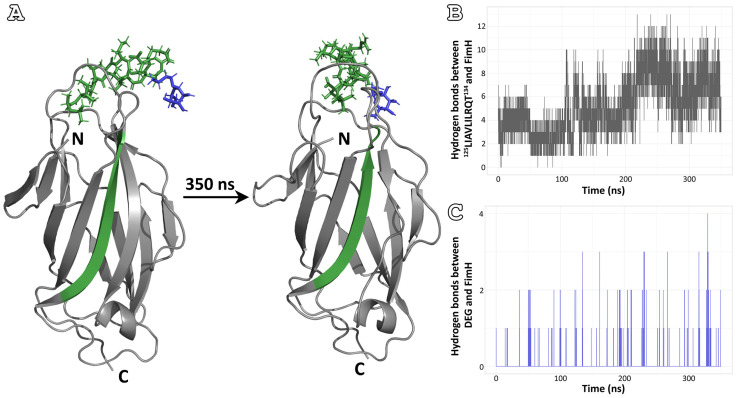
MD simulations of lectin domain-DEG-^125^LIAVLILRQT^134^ complex. (**A**) First (0 ns, left) and last (350 ns, right) frames of MD simulations. ^125^LIAVLILRQT^134^ peptide-analogue is colored green, and DEG is colored blue (N: N-terminal, C: C-terminal). (**B**) The number of hydrogen bonds formed between ^125^LIAVLILRQT^134^ peptide-analogue and FimH lectin domain. More bonds are formed by the end of the simulation. (**C**) The number of hydrogen bonds formed DEG and FimH lectin domain. The number of hydrogen bonds between the two molecules fluctuates during the simulation, with that being zero in most instances.

Analyzing the simulations of the four peptide-analogues, the most promising results were extracted from the ^125^LIAVLILRQT^134^ peptide-analogue. As can be seen from the first and last frames of the simulation, the peptide-analogue interacts with the protein throughout the simulation (Figure 4A). At the same time, DEG is also close to its original position (Figure 4A). However, the plots of the variation in the number of hydrogen bonds formed between the peptide-analogue and the lectin domain and the D-mannose analogue and the lectin domain during the course of the simulation show a clearer picture (Figure 4B,C). The number of hydrogen bonds between ^125^LIAVLILRQT^134^ and FimH appears to increase during the simulation (Figure 4B). In contrast, the hydrogen bonds between DEG and FimH fluctuate (Figure 4C). For the majority of the course of the simulation, these appear to be zero, especially when the number of hydrogen bonds between the lectin domain and the ^125^LIAVLILRQT^134^ peptide-analogue increases significantly.

**Figure 5:**
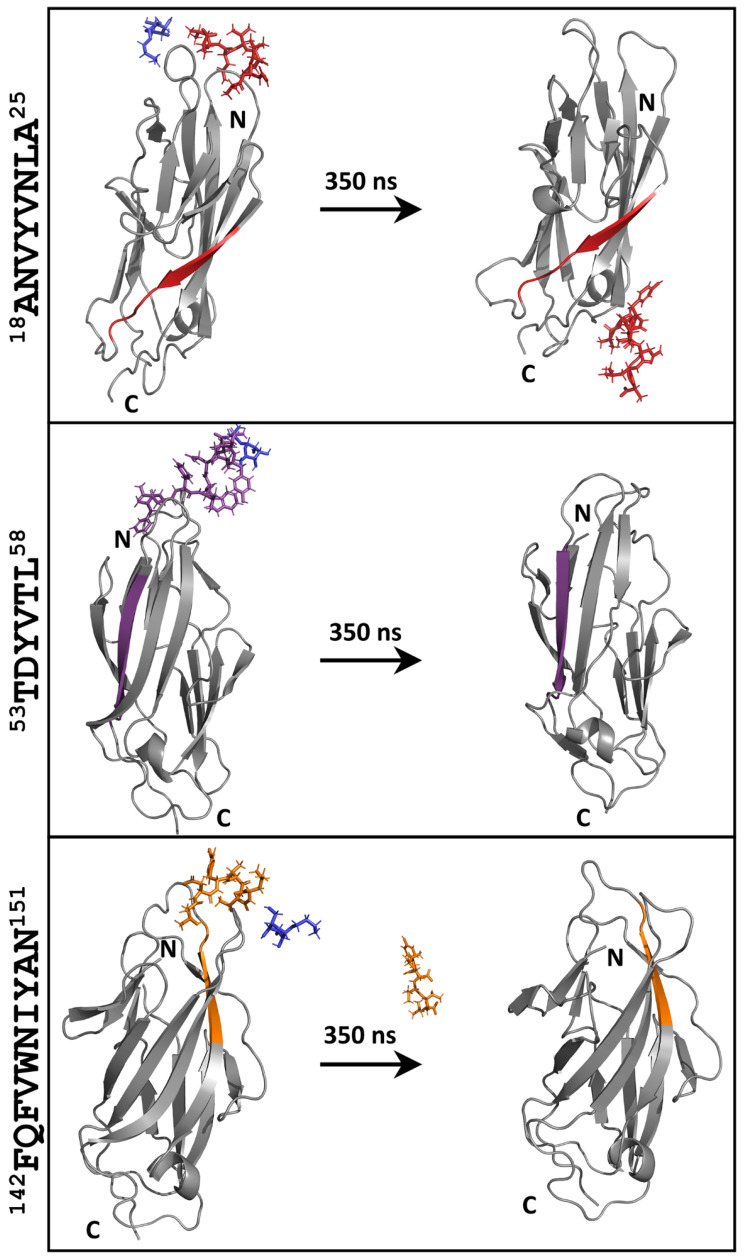
MD simulations of lectin domain-DEG-peptide-analogue complexes. In all three cases, the peptide-analogues moved away from the binding pocket of the lectin domain. ^18^ANVYVNLA^25^, ^53^TDYVTL^58^, ^142^FQFVWNIYAN^151^, and DEG are colored in red, purple, orange, and blue (N: N-terminal, C: C-terminal).

By the comparison of the initial and final frames of the remaining three peptide-analogues, it is evident that at some point in the simulation, they move away from their initial docked position. In the case of ^53^TDYVTL^58^ and ^142^FQFVWNIYAN^151^ peptide-analogues, both the D-mannose analogue and the peptide-analogue have separated from the protein structure (Figure 5). Of interest is the peptide-analogue ^18^ANVYVNLA^25^, which during the simulation, draws away from its initial position and approaches the part of the FimH lectin structure that corresponds to its sequence (Figure 5).

### 3.4. Redundancy of FimH Peptide-Analogues

**Figure 6:**
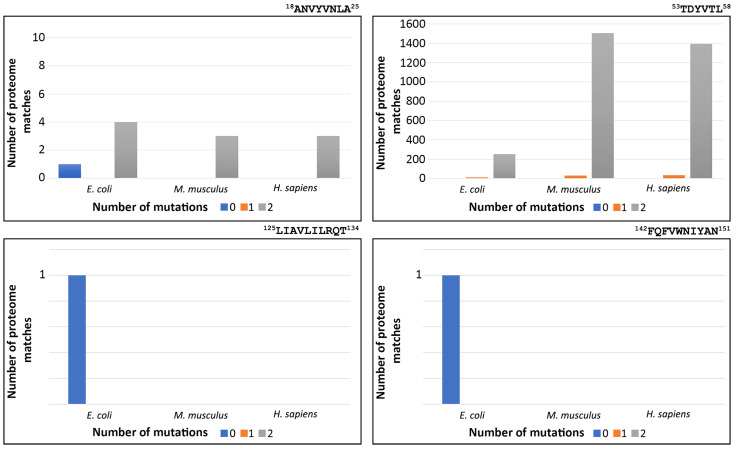
Number of times each FimH peptide-analogue, with zero (0), one (1), or two (2) substitutions, occurs in the proteomes of *E. coli*, *M. musculus* and *H. sapiens*. ^18^ANVYVNLA^25^ peptide-analogue’s sequence is found identical only in the *E. coli* proteome. Its sequence, when carrying 2 mutations, is found 4 times in *E. coli* and 3 times in the proteomes of *M. musculus* and *H. sapiens*, respectively. The sequence of ^53^TDYVTL^58^ peptide-analogue can be identified in one protein in the *E. coli* proteome and one protein in the *M. musculus* proteome. On the other hand, its sequence, when it carries one mutation, can be found in all proteomes. Specifically, it has 10 matches in *E. coli* proteome, 29 matches in *M. musculus* proteome and 33 matches in *H. sapiens* proteome. Additionally, when its sequence has two mutations, it displays 252, 1504 and 1394 matches in *E. coli*, *M. musculus* and *H. sapiens* proteomes, respectively. ^125^LIAVLILRQT^134^ peptide-analogue is found only in the sequence of FimH. No mutant form of it was detected in any of the proteomes. Similarly, ^142^FQFVWNIYAN^151^ peptide-analogue is found only in the sequence of FimH.

A factor that should be taken into consideration when suggesting peptide-analogues as therapeutics is their specificity against the selected target. The four peptide-analogues that we study here should recognize only their sequences and only in bacterial cells in order to be successful against UTIs without causing side effects. Therefore, we calculated the number of times each of the four peptide-analogues occurred in three proteomes, particularly that of *E. coli, M. musculus*, and *H. sapiens*. In each case, zero-, one-, and two-amino acid residue mutations were taken into consideration since several single-point mutations still promote self-aggregation, especially when conservative hydrophobic substitutions take place [52,53]. The results of this computational analysis revealed that ^125^LIAVLILRQT^134^ and ^142^FQFVWNIYAN^151^ peptide-analogues are found only in the FimH protein sequence, while none of the examined proteomes carries the sequences with one or two mutations. Additionally, the peptide-analogue ^18^ANVYVNLA^25^ appears identical only in the *E. coli* proteome (Figure 6). Nevertheless, when its sequence carries two mutations, it is found in four *E. coli* proteins. In most of these cases, the mutations are located mainly in the middle of the sequence and usually involve the replacement of the aromatic residue by a hydrophobic one. Regarding the *M. musculus* proteome, when the sequence carries two substitutions, it is located in three proteins. Similarly, in the *H. sapiens* proteome, the sequence of ^18^ANVYVNLA^25^ with two substitutions is present in three proteins. In these two proteomes, the substitutions are mainly located at the ends of the peptide-analogue. In contrast to all other peptide-analogues, ^53^TDYVTL^58^ is found intact in both the *E. coli* and *M. musculus* proteomes. Additionally, when it carries one or two substitutions, it is found in many proteins of all three proteomes (Figure 6).

## 4. Discussion

The emergence of pathogenic multidrug-resistant Gram-negative bacteria is one of the greatest healthcare challenges of the coming decades. As a result, the treatment of bacterial infections is one of the hottest topics in pharmaceutical research. In this work, we suggest that the amyloidogenic potential of the FimH adhesin protein can be exploited for the design of antimicrobial peptides against UTIs.

Both experimental and computational results indicate that the peptide-analogues ^18^ANVYVNLA^25^, ^125^LIAVLILRQT^134^, and ^142^FQFVWNIYAN^151^ can form amyloid-like fibrils, in contrast to ^53^TDYVTL^58^ peptide-analogue, which self-assembles into amorphous aggregates. Hence, the three amyloid-forming peptide-analogues can identify neighboring identical molecules and interact with them, implying that when administered to bacteria, they are likely to find their sequences on the bacterial FimH protein. During translation, the protein has not acquired its final conformation, with its APRs still exposed to the solvent [65], offering interaction sites for the peptide-analogues. Additionally, in the periplasm, FimH is unstable on its own and needs to interact with the chaperone FimC in order to maintain its folding [66,67]. This binary complex is essential for the activation of the biogenesis mechanism of type I fimbriae [68,69]. Thus, interfering with the folding process of FimH will lead to a cascade of events that will prevent the formation of type I fimbriae and consequently reduce adherence and bacterial invasion to host cells.

The potential of amyloidogenic peptides to act as alternative antimicrobial agents has been highlighted by the work of Khodaparast and her colleagues [70]. More specifically, the researchers relied on the notion that protein aggregation is a sequence-dependent process, which is guided and can be induced through small APRs, which are located on proteins, leading to their self-assembly and aggregate formation [71,72,73,74]. Thus, the researchers identified repetitive sequences with an increased tendency to aggregate on the bacterial proteome and based on these regions; they designed peptides that were administered to bacteria. Indeed, some of these peptides could enter the cell and cause extensive protein aggregation in the form of intracellular inclusion bodies, ultimately leading to bacterial cell death [70]. Furthermore, antimicrobial peptides (AMPs)—a diverse group of naturally occurring proteins that are part of the innate immune system of all multicellular organisms [75]—bring a fresh perspective to the application of amyloid-forming peptides in the treatment of bacterial infections. AMPs have structural, biophysical and biological properties similar to those of amyloidogenic proteins since it has been shown that several AMPs self-assemble into well-ordered fibrillar structures with amyloidogenic properties [76,77,78,79]. Additionally, one of the most well-known amyloidogenic proteins, the Aβ peptide—the main component of the amyloid plaques found in Alzheimer’s disease—exhibits AMP-like antimicrobial activity by disrupting membranes [80]. Thus, the amyloidogenic peptides of FimH can be exploited as potential AMPs against UTIs.

Another interesting observation is that one of the peptide-analogues, ^125^LIAVLILRQT^134^, shows an affinity for the mannose-binding pocket of FIMH and displaces the D-mannose analogue from its original position during the simulation. In the last decades, research has focused on a more anti-adhesive approach for the design of effective FimH inhibitors [81]. In that way, the selective pressure that leads to bacteria antibiotic resistance is avoided. Therefore, the ability of ^125^LIAVLILRQT^134^ peptide-analogue to interact with the mannose-binding pocket of the FimH lectin domain can be used to construct a therapeutic agent that can both prevent UPEC from binding to epithelial cells and remove already attached UPEC from epithelial cells. Furthermore, ^125^LIAVLILRQT^134^ peptide-analogue, as well as ^142^FQFVWNIYAN^151^, fulfills another criterion that enhances its potential as an antimicrobial agent. In order to be effective against UTIs as well as other bacterial infections, pharmaceutical molecules must selectively target bacterial cells, while the host cells must remain unharmed. In our case, we analyzed the redundancy of the four selected peptide-analogues in the proteome of *E. coli, M. musculus* and *H. sapiens*. As can be seen in Figure 6, the sequences of ^125^LIAVLILRQT^134^ and ^142^FQFVWNIYAN^151^ can be found identically only in the proteome of *E. coli*, while their sequence with one or two mutations is not identified in any of the three proteomes. Considering that toxicity to mammalian cells is hindering the development of new candidate AMPs [82], the specificity of these two peptide-analogues for the FimH protein might prevent non-specific interactions with other proteins. However, further experiments are of vital importance to ensure that, indeed, these peptide-analogues are non-toxic for the host cells.

The present study’s results pave the way for a multitude of different studies that will exploit FimH amyloidogenic potential. One promising approach involves the study of the peptide-analogues corresponding to the FimH pilin domain and their effect on the interactions between the pilin and lectin domains. The interaction between these domains is crucial in the transition from low-affinity to high-affinity binding, and as such, it presents an exciting field of study. Our primary future goal is to focus on investigating the potential toxicity and pharmacokinetics of the peptide-analogues *in vivo*, as well as their efficacy in treating bacterial infections in animal models. In summary, the study’s findings highlight the potential for further research into developing novel therapies for bacterial infections using amyloidogenic peptides.

## 5. Conclusions

In summary, the FimH protein has at least three aggregation-prone regions in its lectin domain. From the experimentally studied peptide-analogues, ^125^LIAVLILRQT^134^ seems the most promising one for the development of therapeutic agents against UTIs. This peptide-analogue has the tendency to self-assemble into amyloid-like fibrils, while its sequence is found exclusively in the *E. coli* proteome. Additionally, according to the results of the MD simulations, it is the only peptide-analogue that shows an affinity for the mannose-binding pocket. Therefore, its properties make it the most suitable candidate. Finally, harnessing the amyloidogenic properties of the bacterial sequences could help antimicrobial drug development enter a new era.

## Figures and Tables

**Table 1:** Bands observed in the ATR FT-IR spectra obtained from thin hydrated films produced by ^18^ANVYVNLA^25^, ^53^TDYVTL^58^, ^125^LIAVLILRQT^134^ and ^142^FQFVWNIYAN^151^ solutions, respectively, and their relative assignments.

Bands (cm^−1^)				Band Assignments
^18^ANVYVNLA^25^	^53^TDYVTL^58^	^125^LIAVLILRQT^134^	^142^FQFVWNIYAN^15^	
1136	1138	1136	1134	TFA
-	-	1184	1182	TFA
1205	1203	1205	1203	TFA
1242	-	-	-	β-sheet (amide III)
1516	1516		1516	Tyrosine
1549	1550	1543	1550	β-sheet (amide II)
-	1614			β-sheet (amide I)
1631	1643	1630	1632	β-sheet (amide I)
1676	1676	1670	1665	TFA *

* TFA: trifluoracetic acid, a component of peptide synthesis.

## Data Availability

Data available within the article or Supplementary Materials.

## Supplementary Materials

The following supporting information can be downloaded at: https://www.mdpi.com/article/10.3390/pharmaceutics15031018/s1, Supplementary File S1: Supplementary Figures, Figure S1. Amyloidogenic potential of mature FimH (grey line), Figure S2. Results of MD simulations for FimH lectin domain and butyl alpha-D-mannopyranoside (DEG), Figure S3. Number of intermolecular hydrogen bonds between FimH lectin domain and ^18^ANVYVNLA^25^, ^53^TDYVTL^58^ and ^142^FQFVWNIYAN^151^ peptide-analogues (grey diagrams), respectively, as well as between FimH lectin domain and DEG (blue diagrams), over time; Supplementary File S2: Thioflavin T (ThT) kinetic assay, Figure S4. ThT fluorescence emission spectrum of each individual FimH peptide-analogue over a period of 70 h. Reference [22] is cited in supplementary file S1.

## Abbreviations

UTIs, Urinary Tract Infections; UT, Urinary Tract; UPEC, uropathogenic *Escherichia coli*; APRs, Aggregation-Prone Regions; MD, Molecular Dynamics; TEM, Transmission Electron Microscopy; ATR FT-IR, Attenuated total reflectance Fourier-transform infrared spectroscopy; TFA, Trifluoracetic Acid; AMPs, Antimicrobial Peptides
