# Supplementary material for: Bacterial Lectin FimH and Its Aggregation Hot-Spots: An Alternative Strategy against Uropathogenic Escherichia coli
Source: Pharmaceutics. 2023 Mar 22;15(3):1018. doi: 10.3390/pharmaceutics15031018 (PMC10058141; doi:10.3390/pharmaceutics15031018)
Supplement: Supplementary file 1 [file pharmaceutics-15-01018-s001.zip › Supplementary_File_1.pdf]

Bacterial lectin FimH and its aggregation hot-spots: an alternative strategy against uropathogenic *Escherichia coli*

Georgia I. Nasi[1], Konstantina I. Georgakopoulou[1,a], Marilena K. Theodoropoulou[1], Nikos C. Papandreou[1], Evangelia D. Chrysina[2], Paraskevi L. Tsiolaki[1,b] and Vassiliki A. Iconomidou1,*

[1] Section of Cell Biology and Biophysics, Department of Biology, School of Sciences, National and Kapodistrian University of Athens, Panepistimiopolis, Athens 157 01, Greece

[2] Institute of Chemical Biology, National Hellenic Research Foundation, 48 Vassileos Constantinou Avenue, 11635 Athens, Greece

*** Correspondence to:** Vassiliki A. Iconomidou

Section of Cell Biology and Biophysics, Department of Biology, School of Sciences, National and Kapodistrian University of Athens, Panepistimiopolis, Athens 157 01, Greece.

Phone: +30-210-7274871, Fax: +30-210-7274254, email: veconom@biol.uoa.gr

[a] Present Address: Swiss Institute for Experimental Cancer Research (ISREC), School of Life Science, École Polytechnique Fédérale de Lausanne (EPFL), Lausanne, Switzerland

[b] Present Address: Department of Biochemistry and Biophysics, University of California, San Francisco, California 94143-2240

Supplementary File 1:

Supplementary Figures

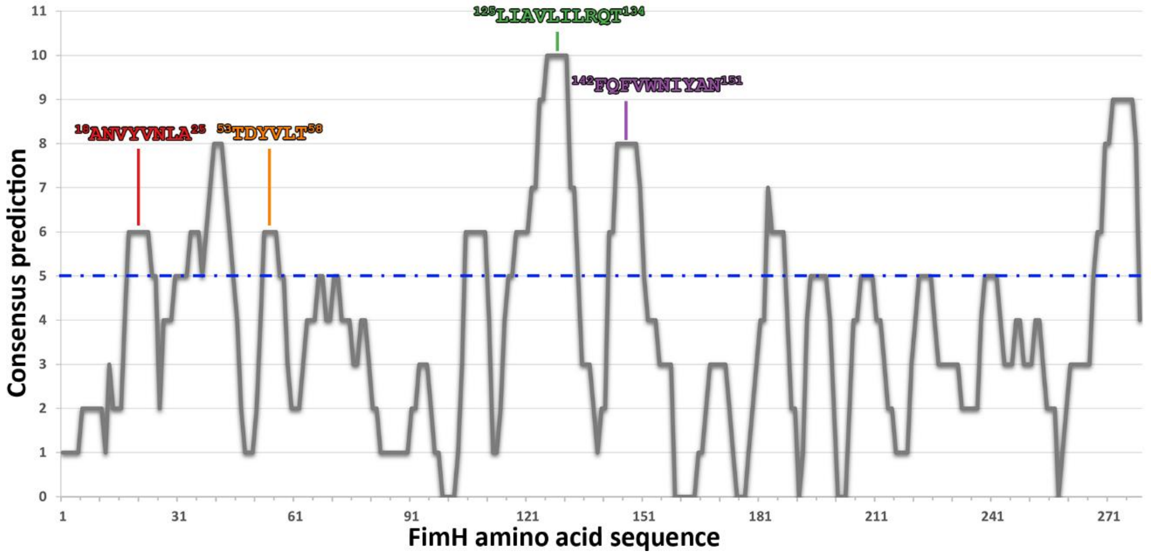

Figure S1. Amyloidogenic potential of mature FimH (grey line). Regions with increased aggregation propensity are observed along the entire length of FimH amino acid sequence. The horizontal axis represents the amino acid sequence, while the vertical axis corresponds to the number of individual prediction algorithms used by AMYLPRED2 (Tsolis et al., 2013). The blue dotted line marks the 5-method default threshold of AMYLPRED2. The sequence of the selected "aggregation-prone" segments for experimental study is highlighted with red (^{18}ANVYVNLA^{25}), orange (^{53}TDYVTL^{58}), green (^{125}LIAVLILRQT^{134}) and purple (^{142}FQFVWNIYAN^{151}).

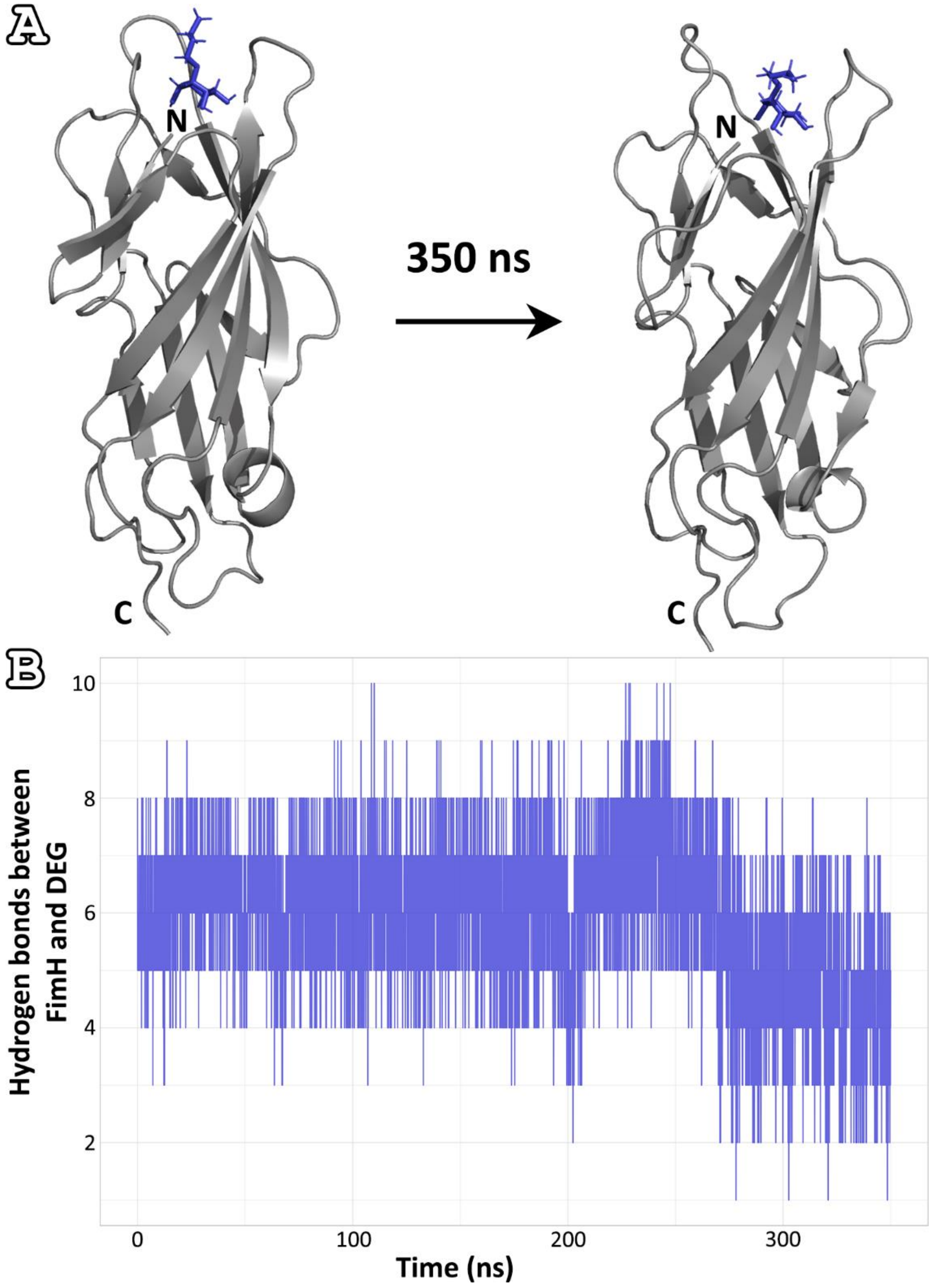

Figure S2. Results of MD simulations for FimH lectin domain and butyl alpha-D-mannopyranoside (DEG). (A) First (0 ns, left) and last (350 ns, right) frames of MD simulations. The DEG molecule (colored in blue) retained its position at the binding pocket until the completion of the 350 ns simulation (N: N-terminal, C: C-terminal). (B) Hydrogen bonds between FimH and DEG. The DEG molecule interacts with the FimH lectin domain throughout the whole simulation.

^{18}ANVYVNLA^{25}

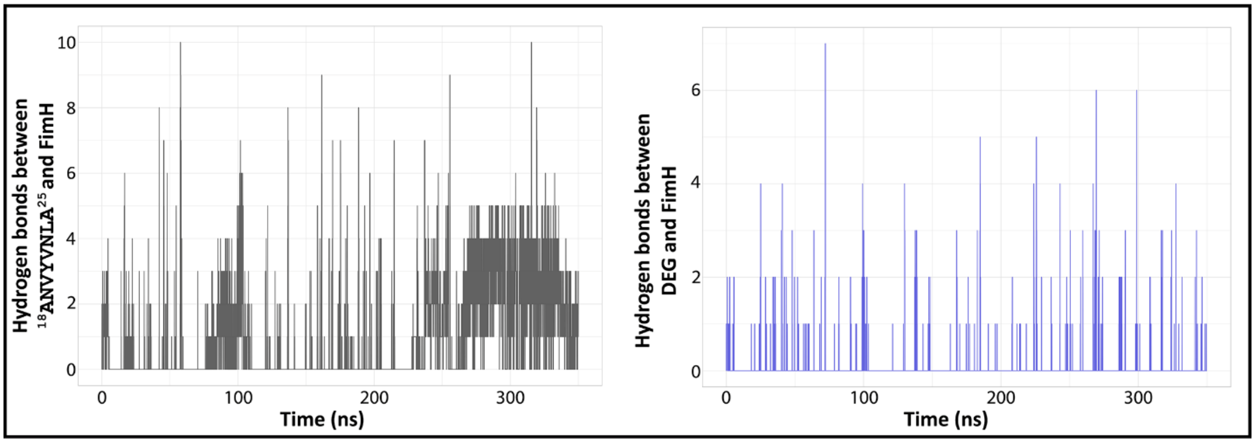

^{53}TDYVTL^{58}

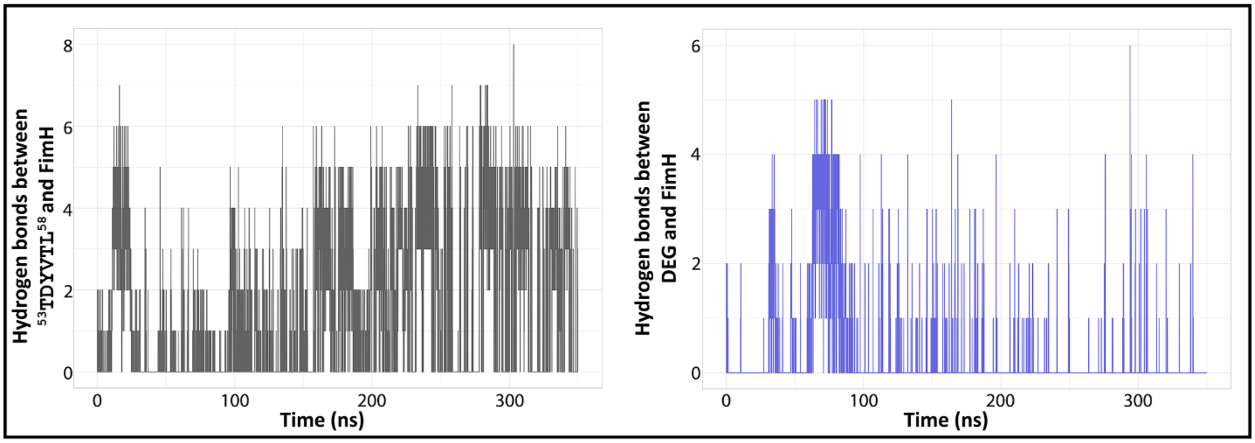

^{142}FQFVWNIYAN^{151}

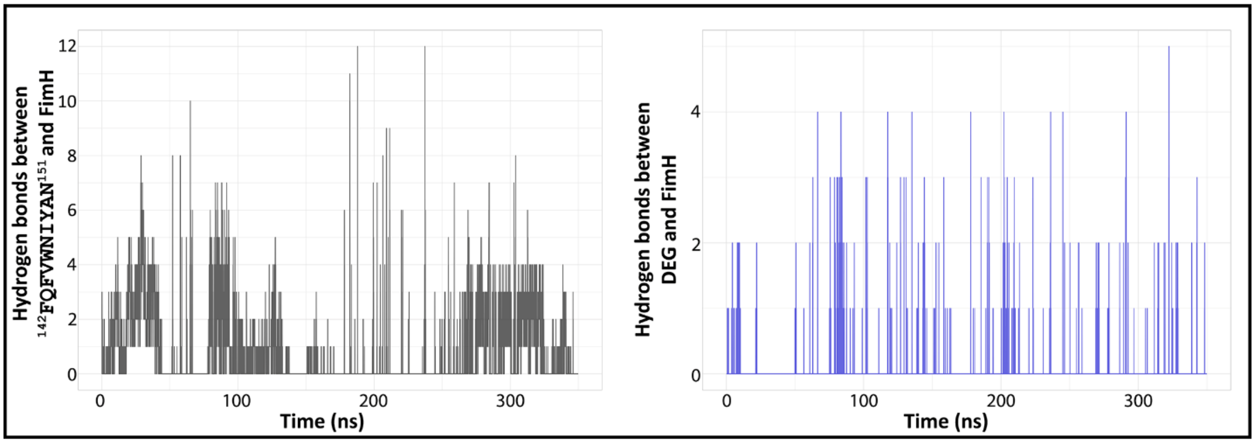

Figure S3. Number of intermolecular hydrogen bonds between FimH lectin domain and ^{18}ANVYVNLA^{25}, ^{53}TDYVTL^{58} and ^{142}FQFVWNIYAN^{151} peptide-analogues (grey diagrams), respectively, as well as between FimH lectin domain and DEG (blue diagrams), over time. The number of hydrogen bonds between the FimH lectin domain and these three peptide-analogues

has large fluctuations during the simulation. Also, in many instances, it reaches zero, contrary to what was observed in the case of ^{125}LIAVLILRQT^{134}peptide-analogue (Figure 4B). Similar fluctuations are observed in the number of hydrogen bonds between the FimH lectin domain and DEG.

Reference

Tsolis, A.C., N.C. Papandreou, V.A. Iconomidou, and S.J. Hamodrakas. 2013. A consensus method for the prediction of 'aggregation-prone' peptides in globular proteins. *PloS one*. 8:e54175.
