# Supplementary material for: Bacterial Lectin FimH and Its Aggregation Hot-Spots: An Alternative Strategy against Uropathogenic Escherichia coli
Source: Pharmaceutics. 2023 Mar 22;15(3):1018. doi: 10.3390/pharmaceutics15031018 (PMC10058141; doi:10.3390/pharmaceutics15031018)
Supplement: Supplementary file 1 [file pharmaceutics-15-01018-s001.zip › Supplementary_File_2.pdf]

Bacterial lectin FimH and its aggregation hot-spots: an alternative strategy against uropathogenic *Escherichia coli*

Georgia I. Nasi[1], Konstantina I. Georgakopoulou[1,a], Marilena K. Theodoropoulou[1], Nikos C. Papandreou[1], Evangelia D. Chrysina[2], Paraskevi L. Tsiolaki[1,b] and Vassiliki A. Iconomidou1,*

[1] Section of Cell Biology and Biophysics, Department of Biology, School of Sciences, National and Kapodistrian University of Athens, Panepistimiopolis, Athens 157 01, Greece

[2] Institute of Chemical Biology, National Hellenic Research Foundation, 48 Vassileos Constantinou Avenue, 11635 Athens, Greece

*** Correspondence to:** Vassiliki A. Iconomidou

Section of Cell Biology and Biophysics, Department of Biology, School of Sciences, National and Kapodistrian University of Athens, Panepistimiopolis, Athens 157 01, Greece.

Phone: +30-210-7274871, Fax: +30-210-7274254, email: veconom@biol.uoa.gr

[a] Present Address: Swiss Institute for Experimental Cancer Research (ISREC), School of Life Science, École Polytechnique Fédérale de Lausanne (EPFL), Lausanne, Switzerland

[b] Present Address: Department of Biochemistry and Biophysics, University of California, San Francisco, California 94143-2240

Supplementary File 2:

Thioflavin T (ThT) kinetic assay

Thioflavin T (ThT) is a fluorescent probe used to study the fibrillation kinetics of amyloid-forming peptides and proteins. To monitor the aggregation kinetics of the FimH peptide-analogue with ThT fluorescence, firstly, each lyophilized peptide-analogue was dissolved in 1,1,1,3,3,3-Hexafluoro-2-propanol (HFIP; Sigma-Aldrich) at a concentration of 1 mg/mL. Individual peptide solutions were left to dry overnight in a fume hood, at room temperature, until thin peptide-containing films were created. The peptide-containing films were stored at −20 °C. ThT fluorescence measurements were carried out using a Tecan Spark microplate reader at 37 °C. Black 96-well plates with flat, transparent bottoms were used and sealed with microplate covers and the florescence was measured through the bottom. A 444 nm filter was used for excitation and a 484 nm filter for emission. HFIP peptide films were dissolved in dimethylsulphoxide (DMSO) and diluted in PBS (pH 7.4) for a final DMSO concentration of less than 5% v/v. The reaction solutions contained freshly prepared 11 μM disaggregated peptide solutions and 50 μM ThT (Sigma©) in dH_2O. ThT background fluorescence was measured in the absence of peptide solutions. Each experiment was conducted three times. The total duration of the measurements was 72 hours, and the fluorescence readings were collected every 15 min. The visualization of the data was performed using RStudio (package ggplot2).

The results of the experiment indicate that ^{18}ANVYVNLA^{25}, ^{125}LIAVLILRQT^{134} and ^{142}FQFVWNIYAN^{151} peptide-analogues are still able to form amyloid fibrils, while ^{53}TDYVTL^{58} does not form any amyloid fibrils. However, their kinetics differ, with ^{18}ANVYVNLA^{25} and ^{125}LIAVLILRQT^{134} peptide-analogues reaching maximal fluorescence signal after approximately 16 and 20 hours, respectively, and ^{142}FQFVWNIYAN^{151}peptide-analogue taking longer to form amyloid fibrils, reaching maximal fluorescence signal after approximately 70 hours (Figure S4).

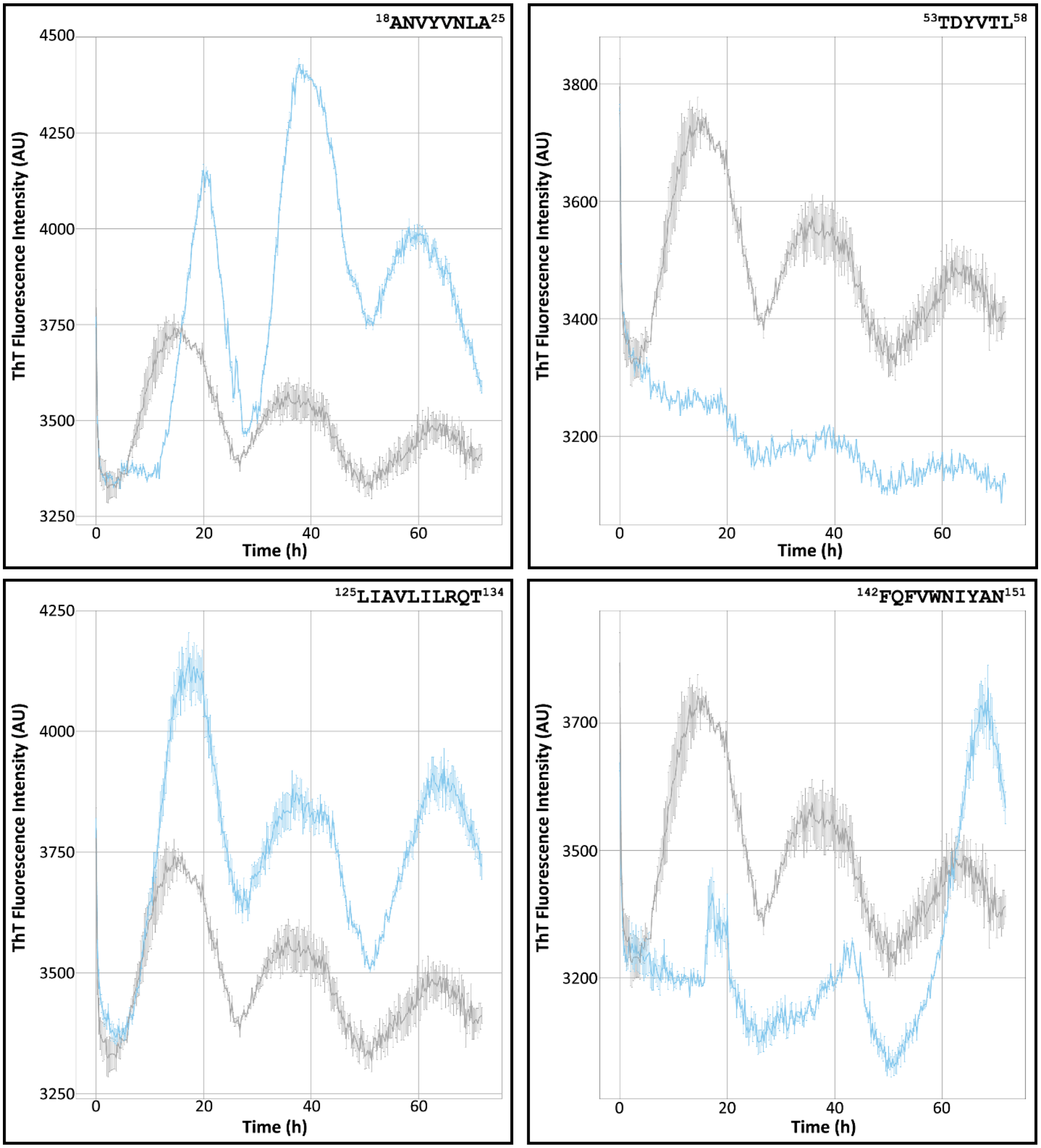

Figure S4. ThT fluorescence emission spectrum of each individual FimH peptide-analogue over a period of 70 hours. The data is not normalized, and values appear as raw data. ^{18}ANVYVNLA^{25}, ^{125}LIAVLILRQT^{134} and ^{142}FQFVWNIYAN^{151} peptide-analogues emit fluorescence higher than ThT background fluorescence after approximately 16, 20 and 70 hours, respectively. On, the other hand, ^{53}TDYVTL^{58} emit fluorescence less than ThT background fluorescence, meaning that it do not form fibrils. All experiments were performed 3 times.
